# Curcumin In Situ Gelling Polymeric Insert with Enhanced Ocular Performance

**DOI:** 10.3390/pharmaceutics12121158

**Published:** 2020-11-28

**Authors:** Hamdy Abdelkader, David Wertheim, Barbara Pierscionek, Raid G. Alany

**Affiliations:** 1 Pharmaceutics Department, Faculty of Pharmacy, Minia University, Minia 61519, Egypt; 2 Department of Pharmaceutics, Faculty of Pharmacy, Deraya University, New Minia City, Minia 61519, Egypt; 3 School of Computing and Information Systems, Faculty of Science, Engineering and Computing, Kingston University London, Penrhyn Road, Kingston upon Thames KT1 2EE, UK; d.wertheim@kingston.ac.uk; 4 School of Life Science and Education, Staffordshire University, College Road, Stoke-on-Trent ST4 2DE, UK; barbara.pierscionek@staffs.ac.uk; 5 Drug Discovery, Delivery and Patient Care Research Group, Faculty of Science, Engineering and Computing, Kingston University London, Penrhyn Road, Kingston upon Thames KT1 2EE, UK; 6 School of Pharmacy, The University of Auckland, Auckland 1023, New Zealand

**Keywords:** curcumin, ocular delivery, polymeric insert, corneal permeability, precorneal residence time

## Abstract

The search for an ocular drug delivery system that could provide long-acting effects without a detriment to the anatomy and physiology of the eye remains a challenge. Polyphenolic compounds (curcumin in particular) have recently gained popularity due to their powerful antioxidant properties; yet curcumin suffers poor stability and water solubility. A conventional eye drop formulation of curcumin in the form of a suspension is likely to suffer a short duration of action requiring multiple instillations. On the other hand, polymeric in-situ gelling inserts offer the prospect of overcoming these limitations. The aim of this study was to prepare, characterize and evaluate in vivo, polymeric, in-situ gelling and mucoadhesive inserts for ocular surface delivery of curcumin. Different types and ratios of biocompatible polymers (HPMC, CMC, PL 127 and PVA) and three plasticizers along with the solvent casting method were adopted to prepare curcumin inserts. The inserts were investigated for their physicochemical characteristics, applicability, and suitability of use for potential placement on the ocular surface. The prepared inserts revealed that curcumin was mainly dispersed in the molecular form. Insert surfaces remained smooth and uniform without cracks appearing during preparation and thereafter. Improved mechanical and mucoadhesive properties, enhanced in vitro release (7.5- to 9-fold increases in RRT_300_ min) and transcorneal permeation (5.4- to 8.86-fold increases in Papp) of curcumin was achieved by selected in-situ gelling inserts compared to a control curcumin suspension. The developed inserts demonstrated acceptable ocular tolerability, enhanced corneal permeability, and sustained release of curcumin along with retention of insert formulation F7 on the ocular surface for at least two-hours. This insert provides a viable alternative to conventional eye drop formulations of curcumin.

## 1. Introduction

The notion of inserting a solid dosage form of ocular pharmaceuticals into the conjunctival sac was proposed decades ago [1,2]. Solid ocular inserts as well as temperature-triggered in situ gels were shown to provide advantages for glaucoma patients over standard eye drops. These advantages include prolonged ocular drug release, greater ocular drug bioavailability and reduction in systemic drug absorption resulting from rapid drainage of eye drops through the nasolacrimal duct, thereby minimizing the number of instillations of eye drops required by patients [1,3,4]. Two ophthalmic products based on ocular inserts, Alza Ocusert^®^ (Pilo-20 and Pilo-40) and Lacrisert^®^, have been produced. Ocusert^®^, a long-acting reservoir antiglaucoma solid insert containing pilocarpine, is no longer commercially available. Although it allowed drug release from the reservoir device over a relatively longer time compared to conventional pilocarpine eye drops, the delivery device was non-biodegradable [5]. Lacrisert^®^, made of hydroxypropyl methyl cellulose, is a biodegradable insert, used for management of dry eye symptoms, albeit drug free.

Limitations of an ocular insert include foreign body sensation, ocular discomfort, and possible expulsion of the insert during sleep along with concerns about cost-effectiveness [2]. These limitations could be overcome by using more favorable materials and bespoke design and dimensions that would minimize foreign body sensation through an improved fit to the conjunctival sac; sufficient malleability to adapt to the ocular surface along with mucoadhesive characteristics to promote prolonged ocular retention [6]. These advantages have been reported with novel ocular films/inserts that have the ability to transform from ‘’solid to gel’’ upon contact with the surface of the eye [7,8]. These novel in situ gelling inserts could be placed in the conjunctival sac where they would swell, gel, and then undergo complete biodegradation. In addition, these inserts could serve an additional purpose, to improve the chemical stability of the auto-oxidizable drug naltrexone hydrochloride [6]. Nevertheless, the performance of these novel inserts on the ocular surface could be further improved.

**Figure 1:**
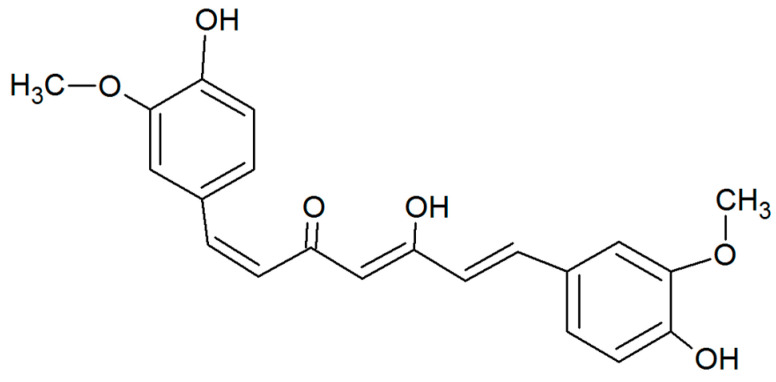
Chemical structure of curcumin.

Bioflavonoids and polyphenolic compounds are two promising classes of naturally occurring compounds that could have applicability for treatment of relevant eye conditions. Bioflavonoids are difficult to formulate due to their poor solubility and inherent chemical instability [9,10]. There has been a growing body of evidence that supports the clinical benefits of curcumin in ophthalmology [11,12,13], a natural phenolic compound derived from turmeric. It has been suggested that curcumin (Figure 1) could enhance corneal wound healing, delay cataract formation, and have beneficial effects in glaucoma, uveitis, and retinal disorders [12,13,14]. These benefits can be attributed to its powerful antioxidant capacity, antiglycation potential as well as inhibiting aldose reductase in the eye [15,16]. The anti-inflammatory effect of curcumin and its potential for treatment of anterior uveitis, dry eye as well as its anti-neovascularization effect have been amongst the recently reported therapeutic benefits for curcumin [17].

Inclusion complexation between hydroxypropyl cyclodextrin and curcumin has been reported in previous research. The complexes showed superior ocular bioavailability and enhanced antioxidant activity in the rabbit cornea [18]. In another study, curcumin was formulated as nanomicelles for intranasal delivery to improve poor solubility and permeability. These nanocarriers demonstrated improved corneal wound healing in diabetic mice [14]. Micellar solubilization of curcumin was achieved using hydrogenated castor oil 40 and octoxynol 40 for treatment of age-related macular degeneration. These novel curcumin delivery nanosystems significantly reduced expression of vascular endothelial growth factor (VEGF) in D407 cell lines [19]. Despite the evidence in support of the potential use of curcumin in the eye, very little has been reported on strategies to improve its ocular performance through enhancement of its corneal permeation and prolonging its precorneal residence time. Prolonging precorneal residence is pivotal to allow clinically meaningful application with improved treatment outcomes [3,20], hence our interest in a polymeric insert that would allow superior ocular performance of curcumin via prolonged precorneal residence.

The aim of this study was to design and develop in situ gelling inserts for enhanced ocular delivery of curcumin. The specific objectives include investigating the characteristics of the formulated inserts, their mucoadhesion properties, in vitro curcumin release, transcorneal curcumin permeability, corneal irritation and finally monitoring their ocular residence time in vivo.

## 2. Materials and Methods

### 2.1. Materials

Curcumin was supplied from TCI (Tokyo Chemical Industry Co., Ltd. Tokyo, Japan). Hydroxy propyl methylcellulose (HPMC), carboxy methylcellulose sodium (Na CMC), glycerol (GLY), polyethylene glycol (PEG 400), triethylacetate 99% (TEC) were supplied by Alfa Aesar, Heysham, England. Polyvinyl alcohol (PVA) molecular weight (MW) 31kDa–50 kDa, 98–99% hydrolysed, Pluronic F127 and cellulose membrane MW cut-off 12 kDa–14 kDa were obtained from Sigma-Aldrich (St. Louis, MO, USA).

### 2.2. Preparation of Curcumin Ocular Inserts

#### 2.2.1. Preparation of Polymer Solutions

Carboxy methylcellulose (CMC) (2.5% *w/w*) polymer solution was prepared by weighing 7.5 g of CMC sodium which was dispersed in 300 mL of fresh deionized hot water at 80 °C using a magnetic stirrer for 2 h. The polymer solution was sonicated for 1 h in a bath sonicator (VWR Ultrasonic International Ltd., Poole, UK) to achieve a final concentration (2.5% *w/w*) of CMC. The CMC polymer solution was allowed to cool at room temperature, and then stored in the fridge at 5 °C until use. Similarly, the polyvinyl alcohol (5% *w/w*) solution was prepared by weighing 15 g of PVA and dispersed in 300 mL of fresh deionized hot water at 80 °C and the procedure was completed as mentioned above for CMC.

Hydroxy propyl methylcellulose (2.5% *w/w*) and Pluronic F127 (PL) (10% *w/w*) solutions were prepared by separately weighing 7.5 g and 30 g of HPMC and PL, respectively, and having these dispersed in 300 mL of fresh deionized cold water over a magnetic stirrer for 2 h.

#### 2.2.2. Preparation of Curcumin Inserts

In situ solid-gel inserts were prepared, as previously mentioned, by adopting the solvent casting method [6]. Specific weights of the prepared polymer solutions, plasticizers (PEG 400, GLY and TEC) and curcumin were selected according to each formula composition (Table 1). The specified quantities were added, mixed and dissolved in a 50 mL volumetric flask (VWR International Ltd., Poole, UK). The concentrations of curcumin and plasticizers in all formulations of the inserts were kept constant depending on the weight of solid polymer in solution. The final mixture was poured into a 12 cm diameter Petri-dish and left to dry for 48 h at room temperature to form polymeric films. Each film was cut into circular discs by using cork borers of different sizes (7 mm and 12 mm in diameter) to fit into the lower conjunctival sac. The final formulations were stored in a re-sealable bag in a cool and dry place until use.

F1 to F3 denote in situ gelling ocular inserts composed of three different sustained release polymers, HPMC, CMC and PL127, respectively, at 1:1 *w/w* of sustained release polymer:film forming polymer (PVP) ratio; F4 to F6 denote in situ gelling ocular inserts composed of three different sustained release polymers using HPMC, CMC and PL127, respectively, at 1:2 *w/w* of sustained release polymer:PVP ratio and F7 to F9 denote in situ gelling ocular inserts composed of different types of plasticisers, GLY-, TEC- and PEG400-plasticised inserts, respectively, using a fixed ratio of 1:1:1:1:1 *w/w/w/w* of PVP:HPMC:CMC:PL 127.

### 2.3. Characterization of Curcumin Inserts

#### 2.3.1. Physicochemical, Mechanical, Morphological, Thermal, Spectroscopic and Mucoadhesive Characteristics

A digital micrometre (Mitutoya, Japan) was used to determine the thickness of 6 curcumin inserts. The six curcumin inserts were individually weighed using a digital balance (Toledo, Switzerland) and results reported as described before [6].

Six curcumin inserts from each ophthalmic film formulation were transferred individually into 10 mL glass vials [6]. Each ocular film was placed in 10 mL NaOH (0.1 M) until it totally dissolved, after which the solution was placed in a sonicator (VWR Ultrasonic International Ltd., Poole, UK) for 10 min until full dissolution. Samples were filtered via a 0.45 µm filter (MILLEX^®^ HA, Merck Millipore Ltd., IRL.) and assayed for curcumin content using a UV spectrophotometer (Jenway 7315, VWR International Ltd., Poole, UK), at 268 nm.

Moisture uptake (%) was determined as previously reported [6]. In brief, six curcumin inserts were placed in a small Petri-dish, then, incubated for three days at a temperature of 40 °C ± 5 °C and relative humidity (RH) of 60% ± 5% in an oven (BINDER, Germany). The sorption (percentage of moisture uptake) was determined according to the following equation (1) [6]:

% Moisture uptake = (W − W_o_)/W_o_ × 100 (1)

W_o_ and W denote weight before and after incubation.

For pH measurements, curcumin inserts were placed in a small Petri-dish. A 600 µL volume of fresh deionized water was placed on the surface of the ocular insert and left to equilibrate for 4–8 min. The pH of each insert was determined as described previously [6].

Determination of mechanical properties [tensile strength, strain (%) and folding endurance]

The prepared curcumin inserts were cut to dimensions of 1 cm × 3 cm (width × length) to fit into the Texture analyser grips. Tensile strength and strain % (% elongation at break) were determined using TA_XT Plus Texture Analyser (Stable. Micro-system Ltd., Surrey, UK). The film thickness was measured with a digital micrometre prior to the measurements. The strain and tensile strength were determined at room temperature in the tension mode. The instrument consisted of two tensile grips, the lower one fixed, the upper one movable. The insert formulations were positioned between two clamps and the distance was calibrated at (20 mm). The ophthalmic inserts were pulled by the upper arm at a rate of 1mm / sec until they broke. The strain % (film break elongation) and tensile strength (mPa) were determined by using software of Exponent Lite version 6.1.4.0 (Stable Micro-System Ltd., Surrey, UK) [6].

Two curcumin inserts were cut into squares of 2 cm × 2 cm and the folding endurance was measured by frequently folding the film strips at the same place until the film ruptured. The number of times that the curcumin insert could be folded at the same location until rupture or up to 400 times without rupture provided the value of folding endurance [6].

Surface morphology and topography studies of the prepared curcumin inserts

The morphological ultrastructure of each ophthalmic film formulation was determined using Scanning Electron Microscopy (SEM). (Zeiss.EVO-50), with 5 KV as the voltage of acceleration. Before analysis, the surface of film was sputtered with gold. Three-dimensional surface imaging of the film was performed using a LEXT OLS4100 (Olympus Corporation, Tokyo, Japan) laser confocal microscope, conducted using a similar approach to that previously described [21,22].

Samples of curcumin powder and selected curcumin-free and curcumin-loaded inserts were weighed and placed in an aluminium pan. The pan temperature was increased from 40 °C to 400 °C and at a rate of 10 °C/min using a DSC calorimeter (Toledo Metter DSC822e0, Zurich, Switzerland). The instrument was calibrated with indium. Nitrogen was used as a purging gas at a flow rate of 45 mL/min.

Fourier transform infrared (FTIR) study

Fourier transform infrared (FTIR) spectrophotometry (Thermo Scientific Nicolet IS5, Madison, USA) was employed to collect the spectra of curcumin and selected curcumin-free and curcumin-loaded insert formulations. A sufficient quantity (3–4 mg) was placed to make a thin film to cover the diamond window. Data were collected and analysed by Omnic software 8.2, (Thermo Scientific, Waltham, MA, USA). The spectra of FTIR were recorded at an average of 120 scans and the resolution was 2 cm^−1^.

Mucoadhesion Studies Using Excised Bovine Conjunctival Membrane

Mucoadhesive characteristics of all in situ gelling inserts were explored using a TA_XT Plus_Texture Analyser (Stable Micro-System LTD., Surrey, UK) set at adhesion mode. Bovine conjunctivae were isolated from eyes freshly obtained from a local abattoir. The freshly obtained bovine conjunctiva was isolated with a scalpel blade and spread on a tissue holder, which was subsequently placed in a glass beaker filled with PBS at pH 7.4 at (35 °C ± 0.5 °C). PBS solution was positioned underneath the tissue surface. Cut pieces of the tested insert formulations of 10 mm diameter were attached to the probe using a double adhesive tape. The conjunctiva was moisturised through pipetting (200 µL) of phosphate buffered saline on the attached area. The probe holding the curcumin insert was brought into contact with the surface of bovine conjunctiva at a rate of 0.1 mm/s. The time of contact was set as 2 min and the applied force was 5000 mN. The force needed to detach the curcumin insert from the surface of conjunctiva was recorded and expressed as the force of adhesion (mN). The distance of de-bonding (mm) was the distance moved by the instrumental probe to detach the curcumin insert from the surface of conjunctiva. The area under the detachment force vs. distance curve was recorded as the work of adhesion (mN.mm).

#### 2.3.2. In Vitro Release Studies

In vitro curcumin release was measured using an incubator shaker at 35 °C ± 0.5°C and a speed of 100 strokes per minute. The circular insert (12 mm diameter) was totally immersed in a 50 mL beaker that was filled with PBS (pH 7.4) containing 0.1% of Tween 80. A sample of 1ml was withdrawn from the medium every 30 min and an equal volume of fresh medium was introduced to keep the volume constant. A curcumin suspension (0.1%) was used as the control formulation. A sample of 1 mL was transferred to cellulose membrane tubing and immersed in the release media (50 mL PBS containing 0.1% Tween 80). An aliquot of 0.5 mL was taken from each withdrawn sample and diluted with 0.5 mL of 1 M NaOH. The absorbance was measured by UV spectrophotometer (Spectronic Genesys^®^, with Winspec Software, Spectronic, USA) at 268 nm. The analytical method used was validated according to ICH guidelines (results not shown). This experiment was performed in triplicate.

To elucidate the drug release mechanism from the developed curcumin inserts, the release data was fitted to the Korsmeyer–Peppas Equation (2) [23].

*M_t_*/*M_∞_* = *kt^n^* (2)

where *M_t_/M_∞_, t, k* and *n* are the fraction of drug released at time t, the kinetic constant and the release exponent, respectively.

#### 2.3.3. Ex Vivo Permeation Using Porcine Corneal Models

The ex-vivo permeation of curcumin was studied using excised porcine corneae. The excised corneae were dissected and washed with fresh PBS solution as mentioned in a previous study [24].

The transcorneal permeation study was conducted using a Franz-diffusion cell giving a 1.7 cm^2^ diffusion area at 35 °C ± 0.5 °C (Logan Instrumental Corporation, Somerset, NI, USA). The cell consisted of two compartments: a donor (upper compartment) and a receptor (lower compartment). The porcine cornea was placed on the receptor compartment with the corneal epithelium and endothelium facing the donor and receptor compartments, respectively. Each insert was positioned on the corneal epithelium and sealed with parafilm. The receptor compartment (12 mL) was filled by PBS. A sample of 1ml was withdrawn from the receptor compartment every 60 min and an equal volume of fresh medium was replaced to keep the volume constant. The withdrawn samples were diluted with 1 M NaOH and the absorbance measured as previously mentioned. Apparent permeability coefficient (Papp) cm/s was calculated using Equation (3) [25].

*Papp* = ∆*Q*/∆*t*(3600)*ACo* (3)

where: ∆*Q*/∆*t A* is the steady state flux of curcumin across the cornea; *Co* is the initial drug concentration (µg/cm^3^); *A* is the surface area of the cornea exposed to the formulation (cm^2^).

#### 2.3.4. Bovine Cornea Opacity and Permeability (BCOP) Test

The BCOP test was used to assess the corneal irritation potential of curcumin and that of selected curcumin free and curcumin loaded inserts as described before [6,26]. Freshly excised bovine eyes were obtained from a local abattoir. The positive control used was 1M NaOH and the negative control was NaCl (0.9% *w/v*). The positive and negative controls (liquids) were placed onto the cornea where their effect was localized by using silicone O-rings. Insert formulations were placed directly on the bovine corneas. Controls and test formulations were applied for 30 s followed by rinsing with saline (approximately 10 mL). The corneal response (opacity) was monitored and scored as described previously [24,25]

Sodium fluorescein solution at a concentration of 2% *w/v* and pH of 7.4 was applied to assess the corneal epithelium integrity. The fluorescein dye was visualized under an examination lamp with a cobalt blue filter. Depending on the fluorescein permeability, the cornea was given an individual numerical score which defined the level of opacification [26,27].

#### 2.3.5. Antioxidant Tests

Trolox equivalent antioxidant capacity (TEAC) assay

The antioxidant capacity of curcumin and F7 were studied using the TEAC method as previously described [10]. First, 2,2’-azino-bis (3-ethylbenzothiazoline-6-sulphonic acid (ABTS) was generated and Trolox (500 µM) and curcumin (10 µg/mL) suspension and its equivalent F7 were tested. Standard antioxidants, ascorbic acid and glutathione, were used as positive controls and the antioxidant capacity and TAEC were calculated according to Equations (4) and (5):

Antioxidant capacity sample = OD_control_ − OD_sample_ (4)

where OD_control_ and OD_sample_ are the optical density of ABTS after addition of 20 µL of PBS, and the OD of the sample, respectively.

TEAC = Scavenging capacity_sample_/Scavenging capacity_trolox_ (5)

Cupric reducing antioxidant capacity (CUPRAC) test

This method was described in detail in our previous publication [10]. Copper (II) chloride (0.01 M), ammonium acetate (1 M) and ethanol 96% solution of neocuproine (7.5 mM) were prepared. The absorbance of the mixture was measured at 450 nm. TEAC was estimated by dividing the OD of the sample by the OD of Trolox.

#### 2.3.6. In Vivo Ocular Residence Time of the Prepared Insert

Three male New Zealand rabbits were used in this study. The animal study was conducted in accordance with the ethical guidelines of the Scientific Procedures Act 1986 and associated guidelines, EU Directive 2010/63/EU for animal experiments and was approved by the Minia University Animal Ethics Committee (050-019). The rabbits weighed between 1.5 and 2.0 kg. Each rabbit was kept in a separate cage and an optimized curcumin insert (F7) was inserted in the lower conjunctival sac of one eye. The eyes were photographed at predetermined time intervals until there was complete visual disappearance of curcumin insert.

### 2.4. Statistical Analysis

Statistical analyses were undertaken for assessment of % elongation, strain, force, work of adhesion, and transcorneal permeation parameters (apparent permeability coefficient and flux). These was conducted using Graph Pad Prism 6 software, USA, to test for analysis of variance (ANOVA) with the level for statistical significance taken as *p* < 0.05.

## 3. Results and Discussion

### 3.1. Preparation of Curcumin Inserts

Three hydrophilic polymers, HPMC, CMC and PL 127, were investigated with PVA included in all nine formulations to provide film forming properties (23). HPMC and CMC have been reported to enhance film forming properties [6].

While PL 127 has been used for preparation of polymeric solution eye drops with thermo-responsive gelling properties (24); there are no previous reports on its film forming characteristics. Three different plasticizers, glycerol, polyethylene glycol 400 and triethyl citrate, were used at a concentration of 30% *w/w*.

Eight out of nine curcumin insert formulations were successfully prepared using the solvent casting method (Table 2). The criteria of success were the ability of the polymer blend and to cast out of the solvent in the form of a uniform, homogenous and intact polymeric film that could be cut out easily. Formulation F6, composed of PVA and PL 127, failed the abovementioned criteria. All other ocular inserts prepared by this method were pliable and dividable to different shapes (square, circle, or rectangle, etc.) and sizes.

### 3.2. Characterization of Curcumin Inserts

#### 3.2.1. Weight, Thickness, Drug Content, Moisture Uptake, pH Measurements and Mechanical Characteristics of the Prepared Curcumin Inserts

The prepared curcumin inserts had a weight range from 6 mg to 17 mg, thickness of less than 0.3 mm and uniform diameter of 6 mm (Table 2). These curcumin inserts were designed to fit into the conjunctival sac. This is likely to minimise foreign body sensation, hence, offer better patient acceptability leading to improved clinical outcomes compared with conventional eye formulations.

With the exception of F3 and F8, all the prepared curcumin inserts exhibited acceptable content ranging from 90% to 95% of the nominal curcumin content of 1% *w/w*. The marked reduction in content with the PL 127-based insert (F3) and TEC-plasticised (F8) inserts could be attributed to the poor film forming properties and lack of plasticizing characteristics of these inserts. TEC is a hydrophobic plasticiser that does not mix well with other hydrophilic polymers such as CMC and HPMC, even in the presence of PL 127, which has surface active properties [28] that might be able to solubilize/emulsify TEC.

The pH measurements demonstrated values of 7 to 7.5, which were close to the pH of the resident tears (pH 7.4). These results would indicate compatibility of the prepared curcumin inserts with the physiological pH of the eye. Percentage (%) moisture uptake was in the range of 10% to 30%. GLY-plasticized films (F1 to F3), and CMC- and HPMC-containing polymers, showed relatively higher % moisture uptake than those plasticized with PEG 400 and TEC (25). Glycerol (GLY) is known for hygroscopicity and is a polyhydric alcohol that can form hydrogen bonding with water molecules and could increase surface moisture uptake from ambient conditions.

Two mechanical parameters were measured to elucidate the mechanical properties: tensile strength and strain (%) as a measure of elasticity and malleability of the prepared curcumin inserts. Table 2 shows the measured values of tensile strength and strain recorded for the prepared curcumin inserts. The tensile strength and strain (%) measurements recorded were 5.2 to 18.8 MPa, and 22.5% to 80%, respectively. These results were in good agreement with the results of folding endurance (Table 2). The higher the strain (%), the higher the values recorded with folding endurance. Glycerol (GLY)-plasticized curcumin inserts demonstrated high % elongation at break (strain) with values ranging from 33.6% to 80%, while those plasticized with TEC and PEG 400 were significantly lower (*p* < 0.05) (12.5% to 19%). GLY (MW 92) is a low molecular weight, water miscible liquid that is likely to diffuse and associate well with polymer chains. This results in decreasing stiffness and increasing flexibility of polymer chains. Conversely, relatively higher molecular weight PEG (MW 400) and less miscible hydrophobic TEC (MW 276) tended to offer little of these desirable plasticizing effects, compared to glycerol-plasticized curcumin inserts. It is worth mentioning that the greater strain (%) values recorded for GLY-plasticized curcumin inserts (F1–F5 and F7) would provide the improved mechanical properties needed to adapt to the curvature of the eye globe at the administration site and to resist deformation of the insert resulting from blinking and patient handling.

#### 3.2.2. Surface Morphology and Topography Studies

**Figure 2:**
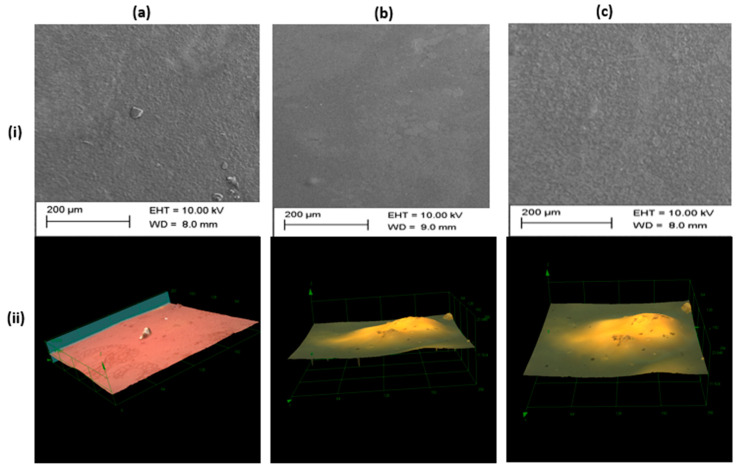
Scanning electron micrographs (**i**) and 3D scanning laser confocal microscopy images (**ii**) for three representative curcumin inserts F1 (**a**), F5 (**b**) and F7 (**c**). The confocal microscope images show views of size 256 × 256 µm from left to right.

Figure 2 shows the surface morphology of three representative curcumin inserts F1, F5 and F7. Scanning electron micrographs (Figure 2(ia–c)) indicate smooth and uniform surfaces without appreciable differences in texture or appearance. Confocal laser scanning microscopy generated 3D-images (Figure 2(iia)) provided new insights into the topography of curcumin inserts. The technique provides superposition of the color image on the 3D surface, thus helping to elucidate the topography. For example, F9 (image not included) showed hill/protrusions on the surface of the insert. This is could be ascribed to residual insoluble curcumin crystals.

#### 3.2.3. Thermal Behaviour (DSC Studies)

**Figure 3:**
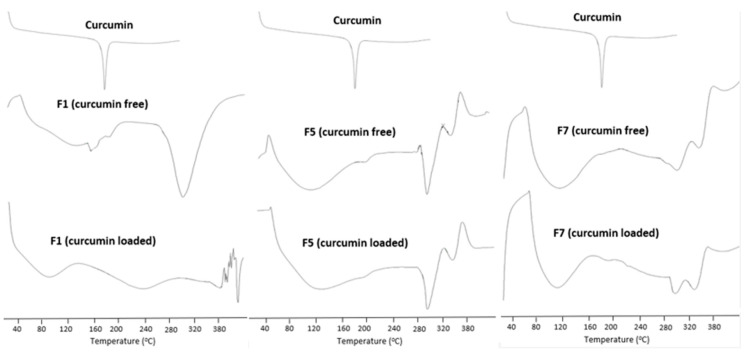
DSC thermograms of three representative curcumin inserts (F1, F5 and F7).

Figure 3 shows DSC thermograms of curcumin and three representative curcumin inserts. DSC is a sensitive and indicative tool of drug crystallinity, melting behaviour and solid state molecular interaction of crystalline drugs. Curcumin, plain inserts (curcumin free) and curcumin loaded inserts were subjected to DSC analysis. Curcumin thermograph showed a sharp endothermic melting peak at 180 °C. The plain F1 (amorphous polymer blend of PVA and HPMC) showed a broad endothermic peak (50 °C to 120 °C) due to elimination of water from the polymers chains as well as another peak > 300 °C which represented polymer decomposition [29]. When curcumin was dispersed and cast within the polymeric matrix F1, there was a complete disappearance of curcumin endotherm. This indicates complete phase transition of curcumin into the amorphous state with possible formation of a solid solution of the drug within the hydrophilic polymeric matrix. A similar behavior was recorded for the other two tested curcumin inserts.

#### 3.2.4. FT-IR Spectroscopy

**Figure 4:**
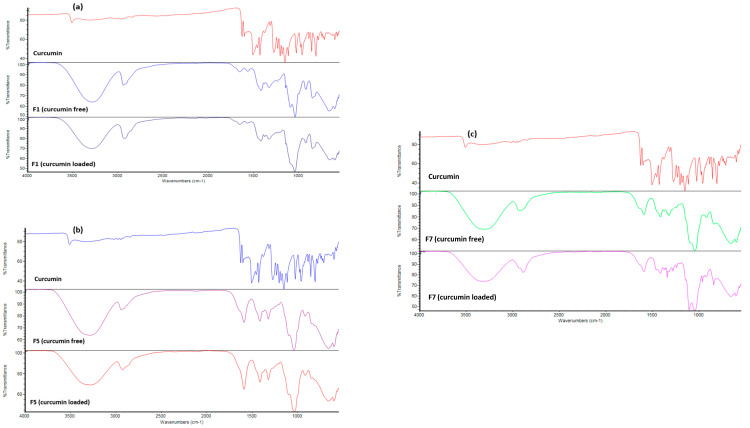
FTIR spectra of three representative curcumin inserts ((**a**) F1, (**b**) F5 and (**c**) F7).

Figure 4 shows representative FTIR spectra for F1, F5 and F7. F5. The FT-IR spectra of PVA and Pluronic F127 demonstrated abroad peak at 3200 cm^−1^ that was indicative of stretching of the OH group and the stretching vibration of CH, C=O and CH from 1600 to 1700 cm^−1^. The FT-IR spectrum of curcumin displayed a typical band involving the OH group of phenol which stretches at 3507 cm^−1^ [18]. Strong bands at 1625 cm^−1^ indicate the stretching vibration of C=O, C=C. The stretching vibration of the aromatic ring is seen at 1601 cm^−1^ [30]. From 1427 to 1455 cm^−1^, the stretching vibration of olefinic –C–H, 1023- cm^−1^ attributed to –C–O–C– is evident. Benzoate C–H trans and cis stretching vibration bands appeared at 976 and 713 cm^−1^, respectively. Disappearance of all characteristic bands of curcumin in F1, F5 and F7 provide further evidence in favor of drug–polymer molecular interaction with subsequent solid solution formation of curcumin in the polymeric matrix of the casted insert. These results are in agreement with those obtained with the DSC studies.

#### 3.2.5. Mucoadhesion Studies

**Figure 5:**
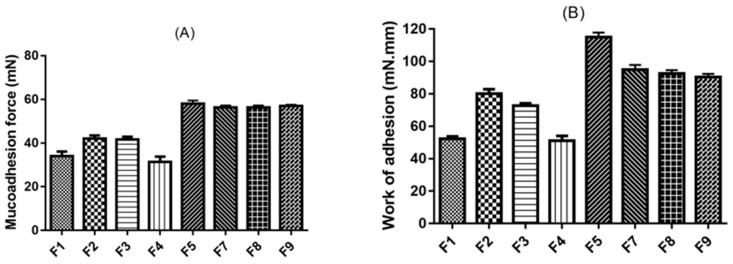
Mucoadhesion forces (**A**) and work of adhesion (**B**) for the prepared curcumin inserts, data represent means ± SD, *n* = 3.

Figure 5 shows two mucoadhesion parameters measured for eight curcumin inserts using excised bovine conjunctiva. Mucoadhesion forces (Figure 5A) and work of adhesion (Figure 5B) measured for curcumin inserts ranged from 36 mN to 63 mN and 52 mN.mm to 118 mN.mm, respectively. All tested curcumin inserts showed sufficient mucoadhesive characteristics [6]. Curcumin inserts F5 to F9 demonstrated significantly greater (*p* < 0.05) mucoadhesion forces and work of adhesion than F1 to F4.

All curcumin inserts were produced using hydrophilic polymers with varying mucoadhesion characteristics. For example, CMC is an anionic polymer with carboxylic groups that can exhibit good adhesion characteristics to the mucopolysaccharide of the mucin secreted by the conjunctival Goblet cells. This is due to the polymer’s hydrogen bonding capacity, and the anionic group’s electrostatic contribution and high molecular weight [6,31]. The higher the CMC concentration in the CMC:PVA ratio, the greater the mucoadhesion and work of adhesion recorded for curcumin inserts. For example, F5 (CMC: PVA was 2:1) demonstrated significantly greater mucoadhesion and work of adhesion than F2 (CMC: PVP was 1:1) (Figure 5). Generally, HPMC has less mucoadhesive characteristics than CMC. This is could be due to its non-ionic nature where a fraction of the free OH groups (hydrogen bonding-forming groups) in the cellulose backbone could be involved in ether linkage with propyl groups [32]. This may explain why F1 (HPMC-based curcumin inserts) had markedly lower mucoadhesive forces and work of adhesion than F2 (CMC-based curcumin inserts).

#### 3.2.6. In Vitro Release and Transcorneal Permeation of Curcumin

**Figure 6:**
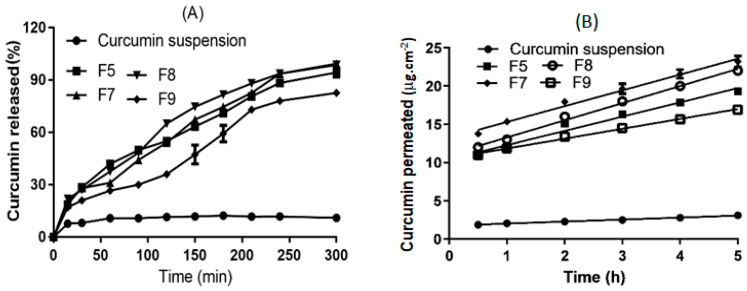
In vitro curcumin release profiles (**A**) and transcorneal permeation profiles of curcumin (**B**) from selected curcumin inserts, data represent means ± SD, *n* = 3.

Figure 6A shows in vitro curcumin release profiles from curcumin suspension (control formulation) and selected curcumin inserts (those that showed superior mucoadhesive characteristics). It was clear that drug dissolution/release from the control suspension (where curcumin existed in an insoluble form at pH 7.4) was extremely low, compared with those from curcumin inserts (the solubilized form of curcumin). The estimated values of RRT_300 min_ revealed 7.5- to 9- fold increases in relative release compared with those from the curcumin suspension (Table 3). This could be ascribed to curcumin being dispersed in a molecular/solid solution form within the polymeric matrix. The release mechanism was Fickian diffusion, as indicated from the release exponent (n) values of around 0.5 (0.44–0.52) where the drug molecules had to diffuse out of the polymeric matrix into the bulk of the release medium.

Figure 6B shows transcorneal permeation profiles of curcumin from the suspension (control formulation) and from some selected curcumin inserts (F5, F7, F8 and F9). Steady state flux and apparent permeability coefficient (Papp) values estimated from transcorneal permeation best fitting straight lines are recorded in Table 3. The steady state flux and P_app_ estimated for the curcumin suspension was as low as 0.27 µg/h. cm^2^ and 0.07 × 10^−6^ cm/sec, respectively. The steady state flux and Papp values recorded for the curcumin inserts were significantly higher (*p* < 0.01) with 5- to 8.3-fold increases and 6- to 8.85-fold increases, compared to curcumin control suspension formulations for the steady state flux and Papp, respectively. These results can be attributed to the higher solubility and faster dissolution rates of curcumin from inserts compared to far less soluble curcumin particles when used as aqueous suspension. This hypothesis is further supported by our DSC results (Section 3.2.4), where we show evidence of the complete phase transition of curcumin into the amorphous state with the likely formation of a solid solution/dispersion of the drug within the hydrophilic matrix of the insert. This would render more curcumin available in the molecular form to dissolve and passively permeate the corneal barrier with which it is in contact.

The cornea is a robust barrier against the permeation/absorption of topically administered drugs [1]. Curcumin is a hydrophobic drug with a log P equal to 3.0. Therefore, curcumin is classified as a Biopharmaceutics Classification Scheme (BCS) II drug. As such, its permeation is mainly dependent on its dissolution rate. The higher the solubility and faster the dissolution rate of this drug, the greater the drug permeability through the corneal barrier. This is demonstrated by the ability of inserts F5, F7, F8 and F9 to improve the RRT300min by 8.7, 9.0, 9.0 and 7.5 folds, respectively, relative to curcumin suspension (Table 3).

#### 3.2.7. BCOP Assay

**Figure 7:**
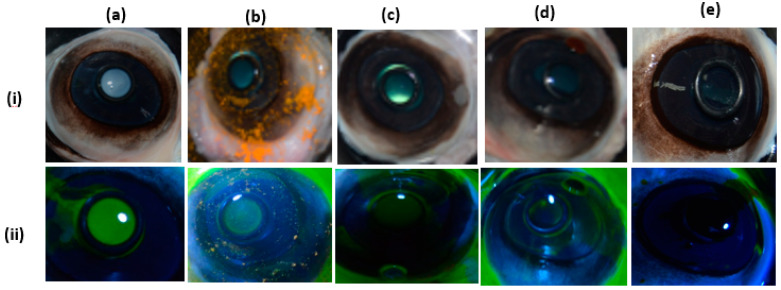
Photo-documentation of Bovine Cornea Opacity and Permeability (BCOP) assay results showing the two assessment criteria, degree of corneal opacity (**i**) and fluorescein permeability (**ii**), used to score. positive control (**a**), negative control (**e**) and test substances / formulations: curcumin powder (**b**), drug free in situ gelling insert (**c**) and curcumin loaded insert (**d**).

**Figure 8:**
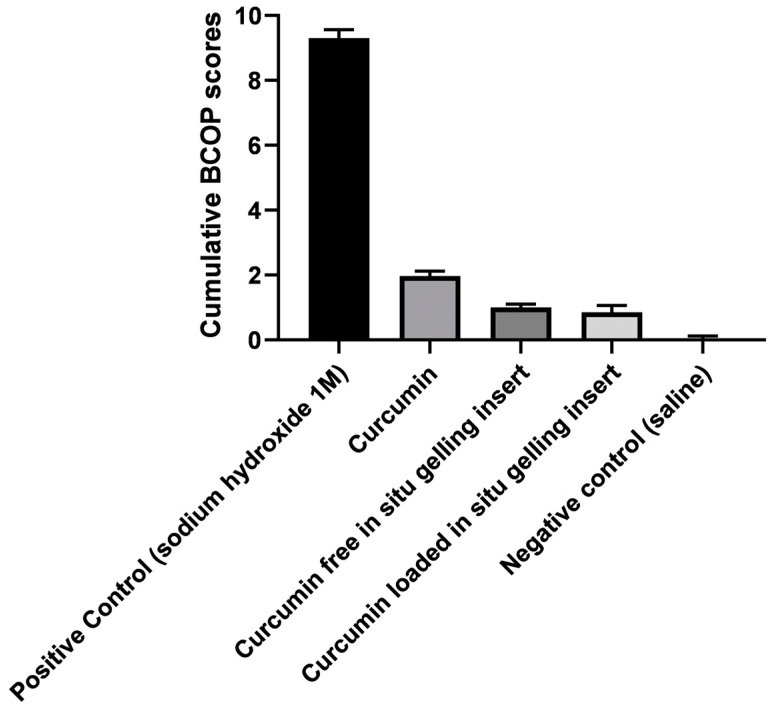
Cumulative BCOP scores for positive and negative controls compared with selected curcumin free and curcumin-loaded inserts, data represent means ± SD, *n* = 3.

Figure 7 shows the micrographic evidence of the results of the BCOP assay for assessment of corneal opacification and fluorescein dye staining caused by curcumin suspension and curcumin inserts. The degree of corneal opacity (upper frame images (i)) and permeability to fluorescein (lower frame images(ii)) responses were photographed and scored. The cumulative scores of the degree of corneal opacity and fluorescein permeability for controls and test substances are shown in Figure 8.

Interestingly, curcumin powder resulted in diffused opacification and confluent fluorescein permeability. Conversely, curcumin insert had slightly lower BCOP scores. This could be explained by the fact that curcumin was dispersed in a molecular state within a polymeric matrix of non-irritant and biodegradable hydrophilic polymers (such as HPMC, CMC and PL127) [28,33], compared to higher local concentrations created by the undissolved and clumped curcumin powder (Figure 7b).

#### 3.2.8. Antioxidant Assays

**Figure 9:**
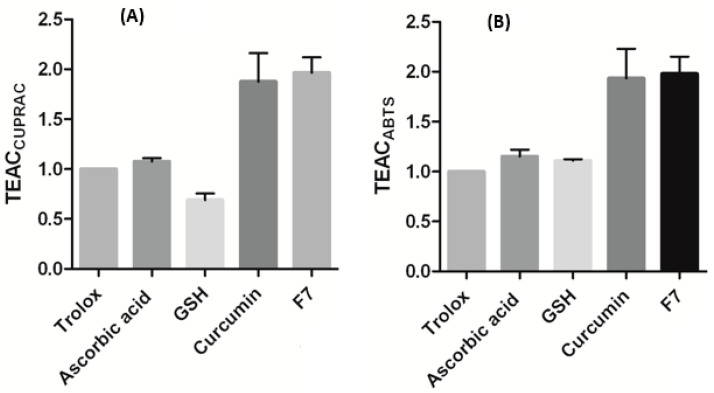
Antioxidant capacity of standard antioxidants (ascorbic and reduced glutathione (GSH)) compared with curcumin powder and curcumin insert (F7) using Cupric reducing antioxidant capacity (CUPRAC) (**A**) and Trolox equivalent assays (**B**), data represent means ± SD, *n* = 3.

To study the effect of formulation process on curcumin antioxidant activities (mainly due to availability of intact polyphenolic functional groups), the antioxidant capacities of curcumin and the curcumin insert F7 were subjected to Trolox and CUPRAC assays. Figure 9A,B shows the antioxidant capacities estimated for ascorbic acid and glutathione as standard controls. These two natural antioxidants are available in the ocular tissue but there is a reduction in glutathione biosynthesis as a consequence of ageing, which has been postulated as a causal factor in age-related nuclear cataract formation [15]. The super antioxidant capacities estimated for curcumin were approximately 2-fold greater than those recorded for ascorbic acid and glutathione. The antioxidant potency is attributed to the intact polyphenolic functional groups in curcumin; this is clearly preserved in the inserts.

### 3.3. In Vivo Ocular Residence Time

**Figure 10:**
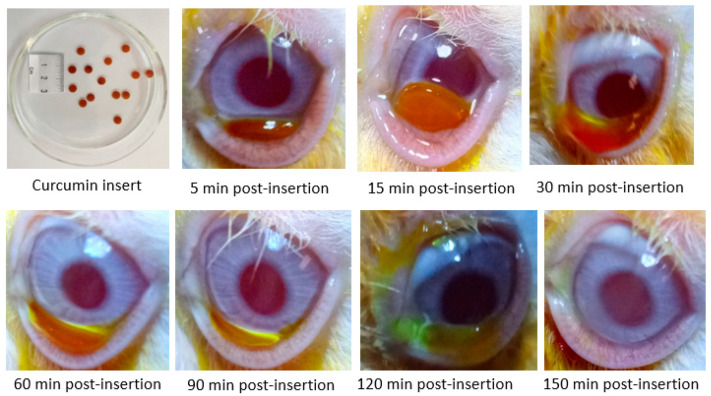
Real-time images demonstrating the in vivo residence/change of curcumin insert (F7) post insertion into the inferior conjunctival cul-de-sac of rabbit eye.

To study the in vivo performance, biodegradability and whether the insert releases the drug at the administration site, curcumin insert (F7) was selected. This selection was informed by the results of the in vitro and ex vivo studies described in previous chapters of this manuscript. Accordingly, insert (F7) was placed in the inferior fornix of the conjunctival sac of the rabbit eye (*n* = 3). The eyes were immediately photographed at predetermined time intervals until complete disappearance of the characteristic brown color of curcumin from the surface of the eye (Figure 10). All three rabbits were alive and with no obvious signs of ill-health or distress at the end of this investigation.

The resident tears hydrated the solid insert and transformed it into a gel. The gel was visible from the 30 min time point and up to 120 min (Figure 10). The tears dissolved the polymeric insert and continuously released the drug for absorption by the ocular surface over the specified time period. This could be mainly ascribed to the mucoadhesive characteristics of the selected curcumin insert (F7). There were no evidence of drug precipitation or phase separation on the ocular surface with drug dissolution, erosion, and complete disappearance of the insert at 150 min. This marked enhancement of the precorneal residence time by F7 would allow less frequent administration of curcumin compared with conventional eye drops that are usually cleared from the ocular surface within minutes. Furthermore, there were no signs of any conjunctival hyperaemia, inflammation, haemorrhage nor any visible changes to the cornea. These observations further confirm the ocular tolerability of these in situ gelling/mucoadhesive curcumin inserts with the results concurring with those of the BCOP assay.

## 4. Conclusions

In situ gelling polymeric inserts loaded with curcumin were successfully prepared using the solvent casting method. Those inserts have the capacity to slowly release curcumin to the ocular surface, enhance its corneal penetration, retain its antioxidant activity and prolong its ocular residence time for over two hours, demonstrating potential for use in the management of ophthalmological conditions where curcumin is indicated. The prepared inserts are sufficiently flexible to enable bending and fitting to the inferior conjunctival fornix and they can be cut into different shapes and dimensions to offer adequate doses of curcumin. The curcumin insert was retained on the ocular surface of the animal model used, for more than two hours, without any signs of ocular surface side effects. These polymeric inserts are promising for the ocular delivery of curcumin where they offer a long-lasting effect compared to solution/suspension eye drops. Further investigations of these inserts, as potential delivery systems for other insoluble and chemically unstable compounds, is warranted.

## Figures and Tables

**Table 1:** Composition and formulation codes of the prepared ocular films.

Formulation Code	F1	F2	F3	F4	F5	F6	F7	F8	F9
Composition									
Curcumin * (mg)	20	20	20	15	15	15	20	20	20
PVA 5% *w/w*	20 g	20 g	20 g	10g	10 g	10 g	10 g	10 g	10 g
HPMC 2.5% *w/w*	40 g			40 g			20 g	20 g	20 g
CMC 2.5% *w/w*		40 g			40 g		20 g	20 g	20 g
PL 127 10% *w/w*			10 g			10 g	5 g	5 g	5 g
GLY	600 mg	600 mg	600 mg	450 mg	450 mg	450 mg	600 mg		
TEC								600 mg	
PEG 400									600 mg

* Curcumin was estimated based on % of total solid weight and kept at a nominal concentration of 1% *w/w.* PVA, HPMC, CMC and PL denote polyvinyl alcohol, hydroxypropyl methylcellulose, carboxy methylcellulose sodium and Pluronic F127, respectively.

**Table 2:** Dimensions, drug content, moisture uptake (%), tensile strength, strain and folding endurance for curcumin insert, data represent mean ± standard deviation (SD), *n* = 6.

Formulation	F1	F2	F3	F4	F5	F6 *	F7	F8	F9
Thickness (µm)	230 ± 12	270 ± 12	280 ± 16	230 ± 12	260 ± 10		270 ± 13	280 ± 18	290 ± 12
Weight (mg)	8 ± 0.2	13.5 ± 0.3	15 ± 0.3	8 ± 0.5	11 ± 0.4		14 ± 0.7	17 ± 0.8	15 ± 0.45
Curcumin content (% *w/w*)	0.95 ± 1.0	0.90 ± 0.2	0.75 ± 6	0.90 ± 0.1	0.90 ± 0.3		0.94 ± 0.1	0.85 ± 0.8	0.95 ± 0.2
Surface pH	7	7.5	7.5	7	7		7	7.5	7.5
Moisture uptake (%)	15 ± 0.2	22 ± 0.7	20 ± 1.2	32 ± 1.5	30 ± 2.0		25.0 ± 14	10 ± 1.4	15 ± 1.3
Tensile strength (MPa)	13 ± 1.5	15 ± 0.7	10 ± 2.6	5.6 ± 1.0	17 ± 1.5		5.2 ± 1.0	17 ± 0.7	18.8 ± 0.9
Strain (%)	61 ± 2.0	29.3 ± 2.5	58 ± 8.0	80 ± 10	13.6 ± 3.6		82 ± 2.4	22.5 ± 1.5	29 ± 3.0
Folding endurance	255 ± 8.4	391 ± 6.0	181 ± 10	205 ± 4.0	360 ± 8.0		380 ± 12	300 ± 7.0	315 ± 5.0

* F6 showed poor film forming properties, and was therefore excluded from further investigation.

**Table 3:** In vitro release and ex vivo permeation parameters for curcumin in situ gelling ocular inserts, data represent means ± SD, *n* = 3.

Formulation Code	In vitro Release Kinetics			Ex vivo Permeation	
	* RRT_300 min_	n	R^2^	Flux (µg.cm^−2^.h^−1^)	P_app_ × 10^−6^ (cm/s)
Curcumin suspension	-	-	-	0.27 ± 0.01	0.07 ± 0.008
F5	8.7	0.52	0.994	1.86 ± 0.05	0.51 ± 0.01
F7	9	0.44	0.98	2.20 ± 0.1	0.60 ± 0.01
F8	9	0.51	0.99	2.24 ± 0.15	0.62 ± 0.02
F9	7.5	0.44	0.99	1.36 ± 0.09	0.42 ± 0.006

* RRT_300 min_ stands for relative release at 300 min; RRT_300 min_ was determined by dividing % cumulative curcumin released from each insert at 300 min by % cumulative curcumin dissolved from curcumin suspension at the same time.
